# Development of a New A^m^–Genome‐Specific Single Nucleotide Polymorphism Marker Set for the Molecular Characterization of Wheat–*Triticum monococcum* Introgression Lines

**DOI:** 10.3835/plantgenome2018.12.0098

**Published:** 2019-11-01

**Authors:** Andras Cseh, Maria Megyeri, Caiyun Yang, Stella Hubbart‐Edwards, Duncan Scholefield, Stephen S. Ashling, Ian P. King, Julie King, Surbhi Grewal

**Affiliations:** ^1^ Agricultural Institute Centre for Agricultural Research, Hungarian Academy of Sciences PO Box 19 2462 Martonvasar Hungary; ^2^ Nottingham BBSRC Wheat Research Centre Division of Plant and Crop Sciences School of Biosciences Univ. of Nottingham Sutton Bonington Campus Loughborough LE12 5RD UK

## Abstract

**Core Ideas:**

- We identified 1247 polymorphic single nucleotide polymorphisms between *Triticum monococcum* and wheat.
- We identified 191 markers validated across all seven chromosomes of *T. monococcum.*
- Detected a *T. monococcum* introgression in leaf‐rust‐resistant lines.

Cultivated einkorn wheat (*Triticum monococcum* L. subsp. *monococcum,* 2*n* = 2*x* = 14, A^m^A^m^) and its wild relative *T. monococcum* subsp. *aegilopoides* are important sources of economically useful genes that can be exploited for wheat (*Triticum aestivum* L.) breeding. Einkorn has excellent resistance to fungal diseases and gene transfer is relatively simple via standard breeding methods. To fulfill the growing demand by modern prebreeding programs for a cost‐effective high‐throughput procedure for accurately detecting introgressed chromosomes or chromosome segments from *T. monococcum* into wheat, we used the Axiom Wheat‐Relative Genotyping Array and developed a set of A^m^ genome‐specific exome‐based single nucleotide polymorphism (SNP) markers suitable for rapid identification of *T. monococcum* chromatin in a wheat background. We identified 1247 polymorphic SNPs between *T. monococcum* and wheat. We identified 191 markers across all seven chromosomes of *T. monococcum* that are also present on an existing *Triticum urartu* Thum. ex Gandil. genetic map and potentially ordered them on the basis of the high macrocollinearity and conservation of marker order between *T. monococcum* and *T. urartu.* The marker set has been tested on leaf‐rust‐resistant BC_3_F_4_ progenies of wheat–*T. monococcum* hybrids. Two markers (AX‐94492165, AX‐95073542) placed on the distal end of the chromosome arm 7AL detected a *T. monococcum* introgression into wheat. The SNP marker set thus proved highly effective in the identification of *T. monococcum* chromatin in a wheat background, offering a reliable method for screening and selecting wheat–*T. monococcum* introgression lines, a procedure that could significantly speed up prebreeding programs.

**Abbreviations**
FISH: fluorescent in situ hybridization
SNP: single nucleotide polymorphism

Wheat (AABBDD, 2*n* = 6*x* = 42) is the most extensively cultivated cereal crop worldwide, supplying the most important food grain source for human nutrition and animal feed. Current wheat production is 752 Tg, with a requirement to reach 858 Tg in 2050 because of the predicted growth of the world's population from 7.6 billion to 9.6 billion (Gupta and Vasistha, 2018).

The tribe *Triticeae* consists of more than 350 annual and perennial species (Ceoloni et al., 2015; Löve, 1984), most of which are important sources of agronomically useful traits that could be exploited for wheat breeding. *Triticum monococcum* L. subsp. *monococcum* (2*n* = 2*x* = 14, A^m^A^m^), commonly known as einkorn, was domesticated from its wild form (*T. monococcum* subsp. *aegilopoides*) about 12,000 yr ago (Heun et al., 1997). *Triticum monococcum* is closely related to *T. urartu* (A^u^A^u^; Johnson and Dhaliwal, 1976), which is the A genome progenitor of durum and bread wheat (Dvořák et al., 1993), with the A^u^ and A^m^ genomes diverging 0.5 to 1 million years ago (Huang et al., 2002). It has been reported that the A^m^ and A^u^ genomes have a high level of gene collinearity (Devos et al., 1995); however, molecular differences have also been found (Wicker et al., 2003). Chromosomal distribution of the sequence identity and comparative analysis of genes between the A subgenome of wheat and those of A genome lineages (*T. urartu* and *T. monococcum*) showed that high sequence similarity and structural conservation are retained, with limited gene loss and chromosomal rearrangement (International Wheat Genome Sequencing Consortium, 2014; Marcussen et al., 2014). High sequence similarity between *T. monococcum* and *T. urartu* also allowed Fox et al. (2014) to map 95% of the 120,911 exome transcripts of *T. monococcum* to the *T. urartu* genome (Ling et al., 2013) successfully.

Today, einkorn is cultivated only marginally to produce traditional or organic products but it harbors many important genes that can be used in wheat breeding (Munns et al., 2012). *Triticum monococcum*, belonging to the primary gene pool of wheat, has excellent resistance to diseases such as leaf rust (*Puccinia triticina*), stem rust, yellow rust, and powdery mildew and several resistance genes [*Lr 10*, (leaf rust resistance); *Sr21*, *Sr22*, and *Sr35* (stem rust resistance); and *Pm25* and *Pm26* (powdery mildew resistance)] have been mapped and transferred to bread wheat (Zaharieva and Monneveux, 2014). Gene transfer from the primary gene pool is relatively simple and is based on standard breeding methods such as homologous recombination, hybridization, and backcrossing (Mujeeb‐Kazi and Rajaram, 2002).

The identification of introgressed chromatin in wheat–ancestral hybrids and backcrossed progenies is a crucial step in the prebreeding process. Fluorescent in situ hybridization (FISH) with repetitive DNA probes made it possible to discriminate between the chromosomes of the *T. monococcum* A^m^ genome and the chromosomes of the A genome of wheat (Badaeva et al., 2015; Megyeri et al., 2017). Nevertheless, this method is limited for identifying small segments of *T. monococcum* chromatin in a wheat background. Therefore, the development of high‐throughput molecular markers covering the entire A^m^ genome is essential for uncovering and detecting new wheat–*T. monococcum* introgression lines.

Genetic maps of einkorn involving restriction fragment length polymorphism markers, isozymes, seed storage proteins, rRNA, and morphological loci have been reported before (Dubcovsky et al., 1996; Taenzler et al., 2002). Simple sequence repeat markers developed in hexaploid wheat have also been mapped on to *T. monococcum* chromosomes, resulting in the construction of an integrated molecular linkage map of the A^m^ genome of *T. monococcum* (Fricano et al., 2014; Hammer et al., 2000; Singh et al., 2007). Genetic linkage maps using Diversity Arrays Technology markers have also been reported for *T. monococcum* (Jing et al., 2009; Marino et al., 2018). Some of these linkage mapping studies in *T. monococcum* compared their genetic maps to the physical map of the *T. urartu* genome and reported that there was a high degree of marker order conservation between A^u^ and A^m^ chromosomes (Fricano et al., 2014; Marino et al., 2018). However, these markers are low‐throughput or have limited success in the wheat background and thus are not suitable for the characterization and identification of wheat–*T. monococcum* recombinant chromosomes in large‐scale pre‐breeding programs.

Here, we present a set of exome‐based SNP markers specific to the *T. monococcum* genome that have proven to be effective in the precise identification of new wheat–*T. monococcum* introgression lines. We used the Axiom Wheat‐Relative Genotyping Array (Affymetrix, Santa Clara, CA) and the allele calling procedure described by King et al. (2017) to identify 1247 polymorphic SNPs between wheat and *T. monococcum.* From among these markers, we selected 191 high‐quality SNP markers that have been validated on wheat–*T. urartu* backcrossed progenies. These could be used as diagnostic markers for detection of *T. monococcum* introgressions in a wheat background.

## MATERIALS AND METHODS

### Plant Materials

One leaf‐rust‐resistant accession of diploid *T. monococcum* subsp. *monococcum* (MVGB1306, obtained from Gene Bank of Martonvasar) was used to produce a wheat–*T. monococcum* interspecific F_1_ hybrid. The hybrid was backcrossed with the wheat parent (cultivar Mv9kr1) to generate BC_1_, BC_2_, and BC_3_ populations (Molnár‐Láng et al., 1996). The BC_3_ plants were self‐fertilized to produce the BC_3_F_1_ generation used for the leaf rust resistance tests. Resistant plants were self‐fertilized three times and the BC_3_F_4_ generation was genotyped by the Axiom Wheat‐Relative Genotyping SNP array. A *T. urartu* genetic map developed previously (Grewal et al., 2018) was used in the present study to select and validate *T. monococcum* chromosome‐specific SNP markers.

### Evaluation of Leaf Rust Resistance

Artificial leaf rust inoculation was performed in a greenhouse with a uredospore suspension on 65 *T. aestivum* × *T. monococcum* BC_3_F_1_ plants during the 2012–2013 growth season. The plants were inoculated at the two‐leaf stage and infection types were recorded on the 10th day after inoculation as described by Stakman et al. (1962).

### Genotyping via an Axiom SNP Array and Selection of *T. monococcum* Genome‐Specific Markers

DNA samples were genotyped by the Axiom Wheat‐Relative Genotyping Array as described by King et al. (2017). The procedure is documented by Affymetrix (https://assets.thermofisher.com/TFS‐Assets/LSG/manuals/axiom_genotyping_solution_analysis_guide.pdf, accessed 12 June 2019). Eight BC_3_F_4_ wheat–*T. monococcum* plants originating from the two leaf‐rust‐resistant BC_3_F_1_ plants (four progeny randomly selected from each parent) and the parental lines (wheat cultivar Mv9kr1 and *T. monococcum*) were genotyped together with the wheat cultivar Paragon, the *T. urartu* parent and 258 samples of the wheat–*T. urartu* backcrossed populations developed by Grewal et al. (2018).

After genotyping with the array, the SNPs were classified into categories as follows: (i) ‘Poly High Resolution’, with at least two examples of the minor allele; (ii) ‘No Minor Homozygote’, with two clusters observed; (iii) ‘Off‐Target Variant’, which had four clusters, one representing a null allele; (iv) ‘Mono High Resolution’, which were monomorphic; (v) ‘Call Rate Below Threshold’, where the SNP call rate was below the threshold but other cluster properties were above the threshold; and (vi) ‘Other’, where one or more cluster properties were below the threshold (Hussain et al., 2017). To select the chromosome‐specific SNPs, the highest quality Poly High Resolution SNPs were used, as they provided three well‐resolved genotype clusters. Flapjack (Milne et al., 2010) was used to remove any SNP markers where (i) either or both parents were clustered as heterozygous calls, (ii) both the wheat and the wild relative parents (*T. monococcum*, *T. urartu*) were clustered together in the same genotype (i.e., no polymorphism) and/or (iii) the parental lines had an undetermined genotype. The polymorphic markers were assigned to *T. monococcum* chromosomes according to information from the genetic map of *T. urartu* (Grewal et al., 2018).

### Comparative Analysis

**Fig. 1:**
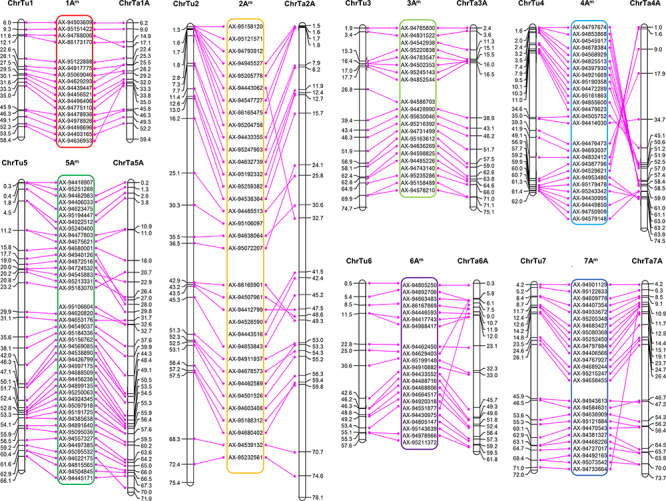
Comparison of physical positions of *T. monococcum* chromosome‐specific single nucleotide polymorphism markers on *T. urartu* chromosomes (Ling et al., 2018) and the A genome of wheat (IWGSC et al., 2018) across the seven homeologous groups. All positions are indicated as 1 × 10^7^ bp.

*T. monococcum* genome‐specific markers, also present on the *T. urartu* map, were used in BLASTN (E‐value: 10^−5^) analysis against the wheat genome reference assembly Refseq version 1 (International Wheat Genome Sequencing Consortium et al., 2018) and the *T. urartu* genome reference assembly (Ling et al., 2018). The results were visualized (Fig. 1) with MapChart version 2.32 (Voorrips, 2002).

## RESULTS

### Response of *T. aestivum* × *T. monococcum* BC_3_F_1_ Seedlings to Leaf Rust Disease

Sixty‐five BC_3_F_1_ wheat–*T. monococcum* seedlings were randomly selected and their response to leaf rust infection was recorded on the 10th day after inoculation (Table 1). Two of the 65 seedlings showed the same immunity as the *T. monococcum* parent, whereas most of the remaining plants (*n* = 55) were very susceptible, similar to the wheat parental line.

### Selection and Validation of *T. monococcum* Chromosome‐Specific SNP Markers

In total, 18,287 SNPs that were polymorphic between *T. urartu* and wheat and distributed across all seven chromosome groups in wheat were included on the Axiom Wheat‐Relative Genotyping Array (Winfield et al., 2016). In the present work, we screened DNA from *T. monococcum, T. urartu,* Mv9kr1 and Paragon wheat, eight BC_3_F_4_ lines originating from the wheat–*T. monococcum* leaf‐rust‐resistant hybrid, and 258 lines obtained from the wheat–*T. urartu* backcrossed population (Grewal et al., 2018). The Axiom ‘SNPolisher’ R package allocated the scores for each of the markers into six cluster patterns (Hussain et al., 2017); however, only the calls classified as Poly High Resolution SNPs (3168 SNPs) represented good quality cluster resolution and were thus included in the genotyping. Of these, 1247 high‐quality SNPs were selected as polymorphic between wheat and *T. monococcum* by Flapjack and were physically mapped to the A genome of wheat via a BLASTN search (Supplemental Table S1; International Wheat Genome Sequencing Consortium et al., 2018). From these 1247 *T. monococcum* SNP markers, 191 were identified as being in common with the *T. urartu* genetic map (Grewal et al., 2018) and were thus selected as a set of validated high‐quality *T. monococcum* chromosome‐specific SNP markers (Table 2). The lowest numbers of SNPs with wheat were detected on homeologous Group 1 (8.9%); homeologous Group 5 (23%) showed the highest number of SNPs.

**Table 1:** Response to leaf rust in 65 *T. aestivum* × *T. monococcum* BC_3_F_1_ plants at the seedling stage.

Infection types	No. of inoculated *T. aestivum* × *T. monococcum* BC_3_F_1_ plants
Very susceptible	55
Moderately susceptible	5
Moderately resistant	1
Very resistant	1
Nearly immune	1
Immune	2

The physical position of the 191 marker sequences was found on the wheat A genome (International Wheat Genome Sequencing Consortium et al., 2018) and on the *T. urartu* genome (Ling et al., 2018) via a BLASTN search (Supplemental Table S2). On the basis of previous work that suggested high synteny and collinearity between *T. monococcum* and *T. urartu* (Fricano et al., 2014; Marino et al., 2018), the 191 SNP markers were tentatively ordered on the *T. monococcum* chromosomes according to their physical position on the *T. urartu* genome (Fig. 1). *Triticum monococcum‐*specific markers also showed macrosynteny with the A genome of wheat (Fig. 1), except in the case of chromosome 4A, which has a pericentric inversion in polyploid wheat that is not found in its diploid progenitor, *T. urartu* (Devos et al., 1995).

**Table 2:** Summary of the number of single nucleotide polymorphisms (SNPs) anchored to *T. monococcum* and *T. urartu* genome on the Axiom Wheat‐Relative Genotyping Array.

	Number of SNP markers	Percentage of total SNP markers	Validated SNPs on the *T. urartu* genetic map	Percentage of total SNPs on the *T. urartu* genetic map
Homeologous Group 1	118	9.5	17	8.9
Homeologous Goup 2	208	16.7	34	17.8
Homeologous Group 3	211	16.9	21	11.0
Homeologous Group 4	155	12.4	27	14.1
Homeologous Group 5	234	18.8	44	23.0
Homeologous Group 6	121	9.7	22	11.5
Homeologous Group 7	200	16.0	26	13.6
Total	1247	100	191	100

From the BC_3_F_4_ progenies of the two leaf‐rust‐resistant plants, eight individuals were randomly selected (four from each parent) and screened with the Axiom Wheat‐Relative Genotyping array, which resulted in the detection of a single *T. monococcum* introgression. Two *T. monococcum*‐specific markers (AX‐94492165 and AX‐95073542), located within 200 bp of each other on the telomeric region of chromosome arm 7AL (Fig. 1), were detected in each of the analyzed plants. The sequences of these two markers were used in a BLASTX search against the wheat genome (International Wheat Genome Sequencing Consortium et al., 2018; http://plants.ensembl.org/Triticum_aestivum/Info/Index, accessed 7 June 2019) to determine any potential candidate genes. However, the immediate flanking regions of the markers did not show any annotated genes.

## DISCUSSION

The genetic diversity of einkorn wheat provides a promising opportunity to improve the resistance of bread wheat against a wide spectrum of fungal pathogens (Zaharieva and Monneveux, 2014). To speed up the gene‐transfer from an alien species into wheat, it is essential to precisely trace the transferred chromosome segments in the progenies. The development of array‐based chromosome‐specific marker sets represent a cost‐effective, high‐throughput solution to accurately identify the introgressed chromosome segments within modern pre‐breeding programs.

The A genome of wheat and those of its diploid relatives (the A^u^ and A^m^ genomes) share a remarkably similar chromosomal gene content, whereby gene sequences are highly conserved, reaching 98% identity (International Wheat Genome Sequencing Consortium, 2014; Marcussen et al., 2014). A previous study by Marino et al. (2018) compared the genetic map of *T. monococcum* and the shotgun assemblies of the *T. urartu* genome (Fox et al., 2014) and the bread wheat genome (Clavijo et al., 2017) and reported a high degree of conservation of marker order between them, since most markers found in the same position or in close proximity in *T. monococcum* were aligned to the same contig in *T. urartu* and bread wheat. The main exception was chromosome 4A, which carries the well‐known pericentric inversion (Devos et al., 1995; Dvorak et al., 2018; Mickelson‐Young et al., 1995), which is consistent with the BLAST results from this study (Fig. 1). In the comparison of *T. monococcum* and the bread wheat genome assembly (Marino et al., 2018), only a few markers for each linkage group (average: 7.2%) were mapped in different bread wheat chromosomes. Grewal et al. (2018) genetically mapped 368 exome‐based SNP markers into seven linkage groups in *T. urartu* and compared them, via BLAST, to the bread wheat genome, reporting that only six markers (1.6%) were located on different A genome chromosomes in wheat.

In the present study, we developed a polymorphic SNP marker set between *T. monococcum* and wheat. By using the recently published wheat reference assembly (International Wheat Genome Sequencing Consortium et al., 2018) and the *T. urartu* genome assembly (Ling et al., 2018) and exploiting the possible macrocollinearity (Fricano et al., 2014; Marino et al., 2018) among *T. urartu* (A^u^A^u^), *T. monococcum* (A^m^A^m^), and the A genome of wheat, we were able to potentially hypothesize the order of the markers within the *T. monococcum* chromosomes (Fig. 1). However, a more accurate approach to identify the order of gene‐specific SNP markers along chromosomes is single‐gene FISH, which can be applied on mitotic metaphase chromosomes. Single‐gene FISH, together with FISH using repetitive sequences, is useful in chromosome identification and allows investigation of chromosome rearrangements and comparative studies on chromosome structure between species with the A genome lineage (Danilova et al., 2014; Said et al., 2018).

Anker and Niks (2001) identified a large number of einkorn accessions that were resistant to leaf rust. Leaf‐rust‐resistant accessions originated in a higher proportion from *T. monococcum* subsp. *monococcum* than from *T. urartu* and *T. monococcum* subsp. *aegilopoides*. Genes from leaf rust resistance have been transferred into wheat from *T. monococcum* subsp. *monococcum* and are located on chromosomes 2A, 3A, and 5A (Dyck and Bartoš, 1994; Kaur et al., 2008; Singh et al., 2007). In this study, we identified two leaf‐rust‐resistant individuals in the progenies of a wheat–*T. monococcum* F_1_ hybrid and indicated that the resistance could potentially be associated with the presence of two *T. monococcum*‐specific SNP markers located on the telomeric region of chromosome arm 7A^m^L. The *Lr20–Sr15–Pm1* resistance locus identified historically in hexaploid wheat that confers resistance to three different fungal wheat pathogens has also been mapped to the distal part of wheat chromosome 7AL (Neu et al., 2002; Sears and Briggle, 1969). Jayatilake et al. (2013) developed expressed sequence tag markers (wri1, wri2, wri3, wri4, and wri5) between the *Lr20/Sr15* locus and the phytoene synthase gene (*Psy‐A1*) and all markers colocated with the *Lr20* gene. These markers are placed on the distal region of chromosome arm 7AL from 724,135,301 bp (wri1) to 726,482,191 bp (wri5) (International Wheat Genome Sequencing Consortium Refseq version 1.0). The *T*. *monococcum* genome‐specific markers presented in this study are positioned between 700,275,508 bp (AX‐94492165) to 700,275,682 bp (AX‐95073542) on chromosome arm 7AL (International Wheat Genome Sequencing Consortium RefSeq version 1.0). Neu et al. (2002) and Jayatilake et al. (2013) have suggested that *Lr20* is in a region where recombination is suppressed. They proposed that this could be caused by an alien introgression or a genetic rearrangement. Our results indicate that a recombination event has taken place on the telomeric region of 7AL chromosome arm, resulting in the introgression of a short *T. monococcum* chromosome segment into the wheat background, carrying an effective leaf rust resistance gene acting in the seedling stage.

The wheat–*T. monococcum* SNP marker set developed and validated in the present study offers accurate, cost‐effective, and high‐throughput detection of *T. monococcum* chromatin in a wheat background and thus significantly speeds up the transfer of valuable traits from a wild relative into bread wheat in modern breeding programs.

### Supplemental Information

Supplemental Table S1. The sequence information of 1247 polymorphic SNPs between *T. monococcum* and wheat.

Supplemental Table S2. BLASTN results detailing the physical positions of the *T. monococcum* specific marker set on the A genome of wheat (RefSeq version 1; International Wheat Genome Sequencing Consortium et al., 2018) and the *T. urartu* psuedomolecules (Ling et al., 2018).

### Conflict of Interest Disclosure

The authors declare that there is no conflict of interest.

## Supporting information

Supplemental File 1

Supplemental File 2
