# Supplementary material for: Development of a New Am–Genome‐Specific Single Nucleotide Polymorphism Marker Set for the Molecular Characterization of Wheat–Triticum monococcum Introgression Lines
Source: Plant Genome. 2019 Nov 1;12(3):180098. doi: 10.3835/plantgenome2018.12.0098 (PMC12810096; doi:10.3835/plantgenome2018.12.0098)
Supplement: Supplementary file 1 [file TPG2-12-180098-s002.pdf]

BLASTN results for *T. monococcum* Am genome specific markers against the physical map of the wheat A genome on the IWGSC RefSeq v1.0 assembly (IWGSC et al., 2018) and the *T. urartu* Tu chromosomes (Ling et al., 2018)

Markers	Chr_Agen	Phy_pos	Chr_Tugen	Phy_pos
name=Chr1Am				
AX-94503609	1A	62206326	Tu1	60478002
AX-95151422	1A	89751245	Tu1	93280187
AX-86173170	1A	149467186	Tu1	120872207
AX-94788006	1A	170600231	Tu1	116057066
AX-95122888	1A	223542281	Tu1	226303995
AX-94629293	1A	252967142	Tu1	295120743
AX-94917775	1A	255444221	Tu1	260582232
AX-95069046	1A	282144295	Tu1	274586498
AX-94439447	1A	291672960	Tu1	300805486
AX-94456521	1A	319818522	Tu1	315684693
AX-94496406	1A	333221487	Tu1	332897478
AX-94636953	1A	338178631	Tu1	535496196
AX-94775110	1A	350349293	Tu1	350097292
AX-94478936	1A	457811595	Tu1	459223146
AX-94978926	1A	462635985	Tu1	462723596
AX-94498696	1A	493107873	Tu1	490962284
AX-94403165	1A	522114869	Tu1	523126701
name=Chr2Am				
AX-95121571			Tu2	13047113
AX-95158120			Tu2	12547018
AX-94945527	2A	14951570	Tu2	16401095
AX-95205778	2A	15962712	Tu2	17307458
AX-94443062	Un	327054188	Tu2	18253511
AX-94547727	2A	17440673	Tu2	18423307
AX-86165475	2A	17656630	Tu2	18496817
AX-94793912			Tu2	16166492
AX-95204758			Tu2	27576048
AX-94433355	2A	79163742	Tu2	73283237
AX-95247903	2A	81784341	Tu2	76915967
AX-94632739	2A	119088544	Tu2	114264512
AX-95192332	2A	123514542	Tu2	125861010
AX-95259382	2A	127205331	Tu2	129564355
AX-94538364	2A	156622873	Tu2	162339230
AX-95072207	2A	240803590	Tu2	364845303
AX-94485513	2A	258347393	Tu2	251089486
AX-94638064	2A	306134021	Tu2	354536228
AX-95106097			Tu2	302854706
AX-94528590	2A	415264731		NO HIT
AX-94443516	2A	423841114	0	NO HIT
AX-94853843	2A	451811748	Tu2	453307603
AX-94412799	2A	475457175	Tu2	435186182
AX-94507961	2A	485791445	Tu2	431570928
AX-86165901	2A	492518272	Tu2	428546019
AX-94911937	2A	529635529	Tu2	513278358
AX-94462589	2A	532856718	Tu2	524651506

AX-94678573	2A	542659166	Tu2	523036025
AX-94501526	2A	552307747	Tu2	530789974
AX-94603408	2A	583170569	Tu2	563721087
AX-94680402	2A	593502125	Tu2	574978571
AX-95188312	2A	597503844	Tu2	571915031
AX-94539132	2A	706919715	Tu2	682567933
AX-95232561	2A	745656548	Tu2	723539628
name=Chr3A				
AX-94785800	3A	24056380	Tu3	19312673
AX-94831522	3A	36490942	Tu3	33660732
AX-94542938	3A	112953496	0	NO HIT
AX-95220838	3A	150722709	Tu3	152646929
AX-95245143	3A	155059111	Tu3	169865353
AX-94783547	3A	159996034	Tu3	163914296
AX-94502353	3A	159996082	Tu3	163914344
AX-94852544	3A	165328732	Tu3	177385819
AX-94586703			Tu3	268484062
AX-94428990	3A	388602444	Tu3	394046104
AX-95630046	3A	430790303	Tu3	433944437
AX-95216392	3A	461791015	Tu3	463006425
AX-94731499	3A	516555053	Tu3	518881960
AX-95163612	3A	575408918	Tu3	569130596
AX-94636269	3A	589600077	Tu3	580801313
AX-94598825	3A	625533016	Tu3	624053807
AX-94485226	3A	638010016	Tu3	628489123
AX-94743140	3A	646125802	0	NO HIT
AX-95235286	3A	659935789	Tu3	648903496
AX-95158489	3A	710274885	Tu3	699171797
AX-94578210	3A	710830615	Tu3	699451747
name=Chr4A				
AX-94853868	4A	589605012	Tu4	15908168
AX-94797674	4A	589604898	Tu4	15908054
AX-94545917	4A	585458309	0	NO HIT
AX-94678384	4A	584273159	Tu4	20068640
AX-94568929			Tu4	24424007
AX-94825513	4A	573889063	Tu4	32091726
AX-94397930	4A	570466105	Tu4	35160017
AX-94472289	4A	525110238	Tu4	94818261
AX-94479623			Tu4	109865069
AX-95161863			Tu4	100607700
AX-94921669	4A	519161347	Tu4	74308278
AX-95190358	4A	512357563	Tu4	79401382
AX-94855600	4A	505793075	Tu4	102936548
AX-94505752	4A	450792527	Tu4	346205765
AX-94414030	4A	347404482	Tu4	350238675
AX-94476473	4A	178844709	Tu4	392501628
AX-94693037			Tu4	418721816
AX-94832412	4A	90420002	Tu4	491102758
AX-94387796	4A	16191593	Tu4	568662851
AX-94529621	4A	9909144	Tu4	576715430

AX-94953480	4A	637895680	Tu4	591675525
AX-95179478	4A	631575949	Tu4	598619685
AX-95243342	4A	629860533	Tu4	600243271
AX-94430995			Tu4	607466288
AX-94579148	4A	611196885	Tu4	614439775
AX-94750909			Tu4	614330902
AX-94449850	4A	609807320	Tu4	613473518
name=Chr5A				
AX-94418907			Tu5	3205447
AX-95251288			Tu5	3205477
AX-94462983	5A	2312249	Tu5	4483446
AX-94406033	5A	13409248	TuUngrouped_contig	5553
AX-94623475			Tu5	18459148
AX-95194447	5A	25703663	0	NO HIT
AX-94922512	5A	37624516	Tu5	44535556
AX-95240400			Tu5	44859782
AX-94477803	5A	109279377	Tu5	111742027
AX-94675621	5A	110373129	Tu5	111930436
AX-94680001	5A	180365847	Tu5	158439600
AX-95183070	5A	206724901	Tu5	231763886
AX-94545883	5A	228886442	Tu5	202207614
AX-95213331	5A	264093219	Tu5	207987162
AX-94872516	5A	269760500	Tu5	189789081
AX-94940126	5A	279563370	Tu5	176589193
AX-94724532	5A	297721300	Tu5	200406713
AX-94653176	5A	317234295	Tu5	310504658
AX-95106604	5A	325952095	0	NO HIT
AX-94620820	5A	327435575	Tu5	299246237
AX-94549037	5A	376098332	Tu5	355969200
AX-95184336	5A	398755170	Tu5	381056211
AX-95156762	5A	442702241	Tu5	419533806
AX-94569085	5A	484437411	Tu5	462731563
AX-94538890	5A	491311590	Tu5	470832923
AX-94426799	5A	491311677	Tu5	470833013
AX-94997175	5A	504873151	0	NO HIT
AX-94688509	5A	535137543	Tu5	500600869
AX-94456236	5A	544573132	Tu5	516736564
AX-94899135	5A	552954126	Tu5	523799967
AX-95250063	5A	552956785	Tu5	523802533
AX-94924345	5A	558692636	Tu5	527959668
AX-95097918	5A	564241428	Tu5	532629708
AX-95191725	5A	575699876	Tu5	540969073
AX-94385638	5A	575699900	Tu5	540969097
AX-94891640	5A	594960176	Tu5	559063375
AX-95095036	5A	601615639	Tu5	564563813
AX-94557327	5A	635502476	Tu5	592271170
AX-94497385	5A	641731122	TuUngrouped_contig	7157
AX-95095532	5A	649843145	Tu5	604110644
AX-94622175	4A	699849740	Tu5	615616987
AX-94815565	5A	660444777	Tu5	615904928

AX-94504845	5A	665477990	0	NO HIT
AX-94445171	5A	673369760	Tu5	628678696
name=Chr6A				
AX-94805250	6A	3289242	Tu6	5092529
AX-94892706	6A	57763779	Tu6	54304000
AX-94663483	6A	58150558	TuUngrouped_contig	8850
AX-86167869	6A	90032550	Tu6	84538345
AX-94446593	6A	107215181	TuUngrouped_contig	6448
AX-94417743	6A	119290095	Tu6	114807946
AX-94988417	6A	119609535	Tu6	115089629
AX-94462450	6A	230706090	Tu6	228191370
AX-94629403	7A	60649459	Tu6	249642445
AX-95199148	6A	322703981	Tu6	306078514
AX-94910882	6A	330043625	0	NO HIT
AX-94433552	6A	456610005	Tu6	426140692
AX-94488716	6A	492793905	Tu6	461729558
AX-94468806	6A	495683926	Tu6	463032795
AX-94664517	6A	517664528	Tu6	486144995
AX-94920318	6A	523935845	Tu6	491548359
AX-94551877	Un	75495746	Tu6	534228387
AX-94430975	6A	564269539	TuUngrouped_contig	4405
AX-94805147	6A	573491342	TuUngrouped_contig	3003
AX-95143639	6A	592278597	TuUngrouped_contig	1223
AX-94978966	6A	594529162	Tu6	550766065
AX-95211372			Tu6	552961348
name=Chr7A				
AX-94901129	7A	42076156	Tu7	41630794
AX-95122633	4A	665502789	Tu7	52167880
AX-94609778	7A	62830945	TuUngrouped_contig	15733
AX-94407354	7A	85153648	Tu7	84010065
AX-94933672	7A	90552082	Tu7	87241034
AX-95205348	7A	109135838	TuUngrouped_contig	64766
AX-94733664	7A	109136179	Tu7	710423933
AX-94683427	7A	117277240	Tu7	116758810
AX-95252450	7A	125194368	Tu7	126375351
AX-95080368	7A	125414671	Tu7	124183277
AX-94797884	7A	144057928	Tu7	141881222
AX-94406566	7A	150926020	Tu7	148169517
AX-94767927	7A	191103215	TuUngrouped_contig	6220
AX-94689244	7A	236549649	Tu7	234987909
AX-95215247	7A	246993158	Tu7	246357680
AX-94658455	7A	263660684	Tu7	261437789
AX-94584631	7A	467348001	Tu7	464881125
AX-94943613	7A	472050035	Tu7	459122156
AX-94638909	7A	542635495	Tu7	535528196
AX-95121884	7A	561529487	Tu7	552830246
AX-94470543	5A	564240610	Tu7	601015620
AX-94381327	7A	645017394	Tu7	629015490
AX-94468226	7A	656942051	Tu7	630501979
AX-94727017	7A	659333272	Tu7	647158471

AX-94492165	7A	700202194	Tu7	684435115
AX-95073542	7A	700202368	Tu7	684435303
